# Approximation of polynomial hulls by analytic varieties: a counterexample

**DOI:** 10.1007/s40627-025-00192-y

**Published:** 2026-01-24

**Authors:** Tobias Harz

**Affiliations:** Universität Bern, Bern, Switzerland

**Keywords:** Polynomially convex hull, Analytic structure, Poletsky discs, Duval–Sibony currents, Primary 32E20

## Abstract

We construct a connected, compact set $K \subset \mathbb {C}^2$ with the following property: there exist points $p \in \hat{K} {\setminus } K$ such that there does not exist a sequence $\{A_\nu \}$ of analytic sets $A_\nu \subset \subset \mathbb {C}^2$ with boundary satisfying $p \in A_\nu$ for every $\nu \in \mathbb {N}$ and $\lim _{\nu \rightarrow \infty } bA_\nu \subset K$. For every point in $\hat{K} {\setminus } K$, we explicitly construct a sequence of Poletsky discs, and we compute the weak limit of the pushforwards of the Green current under these discs.

For every compact set $K \subset \mathbb {C}^n$, let

$$\begin{aligned} \hat{K} \mathrel {\mathop :}=\bigg \{z \in \mathbb {C}^n : |f(z)| \le \sup _K|f| \text { for every } f \in \mathbb {C}[z_1, \ldots , z_n]\bigg \} \end{aligned}$$

denote the polynomially convex hull of *K*. If $A \subset \subset \mathbb {C}^n$ is an analytic set with boundary bA⊂K, then the maximum principle shows that $A \subset \hat{K}$. It is well known from the classical examples of Stolzenberg [10] and Wermer [14] of polynomially convex hulls with no analytic structure that not every point *p* in the additional hull $\hat{K} {\setminus } K$ can be explained in this way. However, in those examples, for each point $p \in \hat{K} \setminus K$ there does exist a sequence of analytic sets $\{A_\nu \}$ with boundary, $p \in A_\nu$, such that the Hausdorff limit $\lim _{\nu \rightarrow \infty } bA_\nu$ is contained in *K*. Also, many results show that $\hat{K} {\setminus } K$ carries an analytic structure in a generalized sense, see, e.g., Rossi’s local maximum principle, and the characterizations of polynomially convex hulls due to Poletsky and Duval–Sibony discussed below.

Drinovec Drnovšek and Forstnerič [3] showed that for any connected, compact set $K \subset \mathbb {C}^n$ that is circular, i.e., invariant with respect to the natural action of the unit circle $\mathbb {T}\subset \mathbb {C}$ on $\mathbb {C}^n$, every point $p \in \hat{K} \setminus K$ admits a sequence of analytic discs containing *p* and whose boundaries converge to a subset of *K*. They also give an example of a non-connected, compact, circular set $K \subset \mathbb {C}^2$ such that not every point in $\hat{K} \setminus K$ admits a sequence of analytic discs as above. Porten [8] adapted this example to show that there exist connected, compact, non-circular sets in $\mathbb {C}^2$ with the same property.

In this note, we will give an example of the following type, that addresses approximation of polynomially convex hulls by arbitrary analytic sets instead of analytic discs.

## Theorem 1

There exists a connected, compact set $K \subset \mathbb {C}^2$ with the following property: there exists $p \in \hat{K} \setminus K$ such that there does not exist a sequence $\{A_\nu \}$ of analytic sets $A_\nu \subset \subset \mathbb {C}^2$ with boundary satisfying $p \in A_\nu$ for every $\nu \in \mathbb {N}$ and $\lim _{\nu \rightarrow \infty } bA_\nu \subset K$ in the Hausdorff metric.

The situation is different if we relinquish control on an arbitrary small part of the boundary $bA_\nu$. Indeed, Poletsky [7] proved the following theorem.

## Theorem

(Poletsky) Let $K \subset \mathbb {C}^n$ be compact. Then $p \in \hat{K}$ if and only if there exists a sequence of holomorphic maps $f_\nu :{\bar{\mathbb {D}}} \rightarrow \mathbb {C}^n$ such that $\lim f_\nu (0) = p,$ and such that the following property is satisfied: for every bounded, pseudoconvex Runge domain Ω containing *K*,  every neighborhood *U* of *K* and every ε>0, there exists $\nu _0 \in \mathbb {N}$ such that for $\nu \ge \nu _0$ one has $f_\nu ({\bar{\mathbb {D}}}) \subset \varOmega$ and $\sigma (\{\zeta \in \mathbb {T}: f_\nu (\zeta ) \notin U\}) < \varepsilon$.

Another characterization of the polynomial hull is due to Duval–Sibony [4].

## Theorem

(Duval–Sibony) Let $K \subset \mathbb {C}^n$ be compact. Then $p \in \hat{K}$ if and only if there exists a positive (1, 1)-current *T* with $p \in {{\,\textrm{Supp}\,}}(T)$ such that $dd^\textrm{c} T = {\tilde{\sigma }} - \delta _p,$ where ${\tilde{\sigma }}$ is a representative Jensen measure for (evaluation at) *p*.

Let us call a sequence $\{f_\nu \}_{\nu =1}^\infty$ of holomorphic maps $f_\nu :{\bar{\mathbb {D}}} \rightarrow \mathbb {C}^n$ as above Poletsky discs (for *K*) centered at *p*, and let us call a positive (1, 1)-current *T* as above a Duval–Sibony current (for *K*) centered at *p*. It was shown by Wold [15] that one can obtain a Duval–Sibony current centered at $p \in \hat{K}$ as the weak limit $T \mathrel {\mathop :}=\lim _{\nu \rightarrow \infty } (f_\nu )_*G$ of the pushforwards of the Green current *G* under a sequence of Poletsky discs centered at *p*. Here, the Green current is defined as

$$\begin{aligned} \langle G,\alpha \rangle = -\int _\mathbb {D}\log |\zeta | \cdot \alpha \quad \text {for all } \alpha \in \mathcal {E}^{1,1}(\mathbb {C}). \end{aligned}$$

In the special case of the compact set *K* of Theorem 1, for every point $p \in \hat{K} \setminus K$ we will explicitly construct Poletsky discs $\{f_\nu \}$ for *K* centered at *p*, and we will compute the limit $T \mathrel {\mathop :}=(f_\nu )_*G$.

## The example

Let $\mathbb {D}\mathrel {\mathop :}=\{\zeta \in \mathbb {C}: |\zeta | < 1\}$ and $\mathbb {T}\mathrel {\mathop :}=\{\zeta \in \mathbb {C}: |\zeta | = 1\}$. Set

$$\begin{aligned} K_1 \mathrel {\mathop :}=\{(\textrm{e}^{2\pi \textrm{i}\theta },w) \in \mathbb {T}\times \bar{\mathbb {D}} : |w| = \theta , \theta \in [0,1]\}. \end{aligned}$$

Then $K_1$ is connected and compact, and $\bar{\mathbb {D}} \times \{0\} \subset \hat{K}_1$. Indeed, applying the maximum principle on the discs $\{\textrm{e}^{2\pi \textrm{i}\theta }\} \times \mathbb {D}(0,\theta )$, $\theta \in [0,1]$ shows that $\mathbb {T}\times \{0\} \subset \hat{K}_1$, and another application of the maximum principle implies that ${\bar{\mathbb {D}}} \times \{0\} \subset \hat{K}_1$.

### Proposition 2

Let $p \in \mathbb {D}\times \{0\}$. There does not exist a sequence of analytic sets $A_\nu \subset \subset \mathbb {C}^2$ with boundary such that $p \in A_\nu$ for every $\nu \in \mathbb {N}$ and $\lim _{\nu \rightarrow \infty } bA_\nu \subset K_1$ in the Hausdorff metric.

### Proof

Assume, in order to get a contradiction, that a sequence $\{A_\nu \}$ with the listed properties exists (see the remark below for the precise definition of analytic sets with boundary). Without loss of generality each $A_\nu$ is purely 1-dimensional, and we claim that there exist a sequence $\{X_\nu \}$ of Riemann surfaces, smoothly bounded open sets $D_\nu \subset \subset X_\nu$, and holomorphic maps $\varPhi _\nu :X_\nu \rightarrow \mathbb {C}^2$ such that $p \in \varPhi _\nu (D_\nu )$ for all $\nu \in \mathbb {N}$ and $\limsup _{\nu \rightarrow \infty } \varPhi _\nu (bD_\nu ) \subset K_1$ [recall that the Hausdorff limit $\lim _{\nu \rightarrow \infty }\varPhi _\nu (bD_\nu )$ coincides with $\limsup _{\nu \rightarrow \infty } \varPhi _\nu (bD_\nu ) \mathrel {\mathop :}=\bigcap _{\nu \ge 1}\bigcup _{\mu \ge \nu }\varPhi _\nu (bD_\nu )$, whenever it exists]. Indeed, let $\varOmega _\nu \subset \mathbb {C}^2$ be open sets such that $A_\nu$ is an analytic subset of $\varOmega _\nu$, and let $\rho _\nu :\varOmega _\nu \rightarrow \mathbb {R}$ be a smooth exhaustion function, $\nu \in \mathbb {N}$. Then, by local finiteness of ${{\,\textrm{Sng}\,}}A_\nu$ and Sard’s theorem, for almost all $t \in \mathbb {R}$ the relative boundary of the set $\tilde{D}_\nu ^t \mathrel {\mathop :}=\{q \in A_\nu : \rho _\nu (q) < t\}$ in $A_\nu$ is a compact smooth manifold contained in ${{\,\textrm{Reg}\,}}A_\nu$. For $\{t_\nu \}_{\nu =1}^\infty \subset \mathbb {R}$ converging to ∞ fast enough, the sets $\tilde{D}_\nu \mathrel {\mathop :}=\tilde{D}_\nu ^{t_\nu }$ satisfy $p \in \tilde{D}_\nu$ for all $\nu \in \mathbb {N}$ and $\limsup _{\nu \rightarrow \infty } b\tilde{D}_\nu \subset K_1$. For every $\nu \in \mathbb {N}$, let $\varPhi _\nu :X_\nu \rightarrow A_\nu$ be the normalization of $A_\nu$, i.e., a finite, proper, holomorphic map from a Riemann surface $X_\nu$ to $A_\nu$ that is biholomorphic above ${{\,\textrm{Reg}\,}}A_\nu$ (see, e.g., Proposition 6.2 in [2]). Then $D_\nu \mathrel {\mathop :}=\varPhi _\nu ^{-1}(\tilde{D}_\nu )$ has the desired properties.

Let (*z*, *w*) denote the coordinates in $\mathbb {C}^2$, and write $p=(z_0,w_0)$. Let $U \subset \mathbb {C}^2$ be a connected and simply connected open neighborhood of $K_1$ such that $z_0 \notin \pi _z(U)$, where $\pi _z$ denotes the projection onto the first variable. Let $L :U \rightarrow \mathbb {C}$ be a holomorphic lift of $\pi _z-z_0: U \rightarrow \mathbb {C}^\times$ through $\exp :\mathbb {C}\rightarrow \mathbb {C}^\times$, i.e., $\exp \circ L = \pi _z - z_0$. For every $\nu \in \mathbb {N}$, the boundary $bD_\nu$ consists of finitely many smooth curves $\varGamma _1^{\nu }, \ldots , \varGamma _{N(\nu )}^{\nu }$, and since $\limsup _{\nu \rightarrow \infty } \varPhi _\nu (bD_\nu ) \subset K_1 \subset U$, there exists $\nu _0 \in \mathbb {N}$ such that for every $\nu \ge \nu _0$ and every $k \in \{1, \ldots , N(\nu )\}$ one has $\varPhi _\nu (\varGamma _k^\nu ) \subset U$. Fix $\nu \ge \nu _0$, and define $f_\nu :X_\nu \rightarrow \mathbb {C}$ as $f_\nu (x) \mathrel {\mathop :}=\pi _z(\varPhi _\nu (x))-z_0$. Since, for every $k \in \{1, \ldots , N(\nu )\}$, the image $\varPhi _\nu (\varGamma _k^\nu )$ is contained in *U*, it follows that $f_\nu = \exp \circ L \circ \varPhi _\nu$ near $\varGamma _k^\nu$, and thus

$$\begin{aligned} \int _{\varGamma _k^\nu } \frac{\textrm{d}f_\nu }{f_\nu } = \int _{\varGamma _k^\nu } \textrm{d}(L \circ \varPhi _\nu ) = 0. \end{aligned}$$

By the argument principle (see, e.g., Theorem 3.1.9 in [13]), this proves that

$$\begin{aligned} \sum _{x \in D_\nu } {{\,\textrm{Ord}\,}}_x(f_\nu ) = \frac{1}{2\pi i} \int _{bD_\nu } \frac{\textrm{d}f_\nu }{f_\nu } = 0 . \end{aligned}$$

Hence $f_\nu \ne 0$ on $D_\nu$, i.e., $\varPhi _\nu (D_\nu ) \cap (\{z_0\} \times \mathbb {C}) = \varnothing$, which contradicts the fact that $p \in \varPhi _\nu (D_\nu )$ for every $\nu \in \mathbb {N}$. □

### Remark

(1) The statement of Proposition 2 holds true with respect to the following general definition of analytic sets with boundary (see, e.g., Sect. 14.3 in [2]): A pure *p*-dimensional analytic set $A \subset \mathbb {C}^n$ is called an analytic set with boundary, if its closure $\bar{A}$ is contained in another analytic set $\tilde{A} \subset \mathbb {C}^n$, and if the following properties hold true:

- The set $bA = \bar{A} \setminus A$ has locally finite (2p-1)-dimensional Hausdorff measure.
- There exists a closed set $E \subset \bar{A}$ of (2p-1)-dimensional Hausdorff measure zero, such that A\E is a $\mathcal {C}^1$-submanifold with boundary bA\E of $\mathbb {C}^n \setminus E$.

(2) Let $K \subset \mathbb {C}^2$ be a compact set. The fact that in general not every $p \in \hat{K} \setminus K$ admits a sequence $\{A_\nu \}$ of analytic sets with boundary such that $p \in A_\nu$ for every $\nu \in \mathbb {N}$ and $\lim _{\nu \rightarrow \infty } bA_\nu \subset K$ in the Hausdorff metric is already implicitly contained in the work of Stolzenberg [11]. Namely, purely 1-dimensional analytic sets with boundary satisfy Stolzenberg’s generalized argument principle (see Theorem 2.5 and the subsequent comments and references in [11]); it is easy to see that validity of the generalized argument principle is a property that is preserved under Hausdorff limits; thus if for *every* point in $\hat{K} \setminus K$ there exists a sequence $\{A_\nu \}$ of analytic sets as above, then *K* satisfies the generalized argument principle; however, there are compact sets in $\mathbb {C}^2$ that do not satisfy the generalized argument principle, see, e.g., 2.15 in [11] (note that the compact set in this example, attributed to Bishop, is a special instance of the set *K* to be defined below), and 29.30 in [1].

We give another variant of the above example, which we will consider in more detail. Let σ denote normalized arclength measure on $\mathbb {T}$, i.e., $\sigma (\mathbb {T}) = 1$. Fix a nonempty open set $I \subset \mathbb {T}$ such that $\sigma (\bar{I}) < 1$ and σ(E)=0, where $E \mathrel {\mathop :}=b_\mathbb {T}I$ denotes the relative boundary of *I* in $\mathbb {T}$, and set

$$\begin{aligned} K \mathrel {\mathop :}=((\mathbb {T}\setminus I) \times \{0\}) \cup (\bar{I} \times \mathbb {T}). \end{aligned}$$

In order to provide an example of a connected, compact set, consider also the special case $I = I_+ \mathrel {\mathop :}=\{\zeta \in \mathbb {T}: {{\,\textrm{Im}\,}}(\zeta ) > 0\}$, and define

$$\begin{aligned} K_2 \mathrel {\mathop :}=((\mathbb {T}\setminus I_+) \times \{0\}) \cup (\{-1\} \times {\bar{\mathbb {D}}}) \cup (\bar{I}_+ \times \mathbb {T}) . \end{aligned}$$

All of the subsequent statements on *K* remain true, with the same proofs, for $K_2$.

### Lemma 3

We have $$\hat{K} = ({\bar{\mathbb {D}}} \times \{0\}) \cup (\bar{I} \times {\bar{\mathbb {D}}})$$.

### Proof

As before, it follows from the maximum principle that $({\bar{\mathbb {D}}} \times \{0\}) \cup (\bar{I} \times {\bar{\mathbb {D}}}) \subset \hat{K}$. For the reverse inclusion, observe first that $\hat{K} \subset {\bar{\mathbb {D}}}^2$. Let $p = (z_0,w_0) \in {\bar{\mathbb {D}}}^2$ such that $z_0 \notin \bar{I}$ and $|w_0|>0$. Since $\mathbb {C}\setminus \bar{I}$ is connected, the set $\bar{I}$ is polynomially convex, and we can find $P \in \mathbb {C}[z]$ such that |P|≤1 on $\bar{I}$ and $|P(z_0)| > 1/|w_0|$. Then $Q(z,w) \mathrel {\mathop :}=P(z)w$ satisfies |Q|≤1 on *K* and |Q(p)|>1. □

Every point $p = (z,w) \in \hat{K} \setminus K$ that lies in $\bar{I} \times {\bar{\mathbb {D}}}$ is contained in an analytic disc attached to *K*, namely the disc $\{z\} \times {\bar{\mathbb {D}}}$. For the remaining points in $\hat{K} \setminus K$, we again have, with the same proof as before, the following result.

### Proposition 4

Let $p \in \mathbb {D}\times \{0\}$. There does not exist a sequence of analytic sets $A_\nu \subset \subset \mathbb {C}^2$ with boundary such that $p \in A_\nu$ for every $\nu \in \mathbb {N}$ and $\lim _{\nu \rightarrow \infty } bA_\nu \subset K$ in the Hausdorff metric.

We will explicitly construct Poletsky discs for *K* centered at any point $p \in \hat{K} \setminus K$. If $p = (z_0,w_0) \in \bar{I} \times {\bar{\mathbb {D}}}$, then we simply take the single disc $f_\nu (\zeta ) \mathrel {\mathop :}=(z_0,\zeta )$. If $p \in \mathbb {D}\times \{0\}$, then we proceed as follows: For every real-valued function $f \in L^1(\mathbb {T})$, let

$$\begin{aligned} P[f](r\textrm{e}^{\textrm{i}\theta }) \mathrel {\mathop :}=\frac{1}{2\pi } \int _{-\pi }^\pi \frac{1-r^2}{1 - 2r\cos (\theta - t) + r^2} f(\textrm{e}^{\textrm{i}\theta }) \,\textrm{d}t \end{aligned}$$

denote its Poisson integral. Then *P*[*f*] is harmonic on $\mathbb {D}$, and extends continuously to all points of $\mathbb {T}$ at which *f* is continuous. Let now $u \mathrel {\mathop :}=-P[\chi _{\mathbb {T}\setminus \bar{I}}]$, where $\chi _{\mathbb {T}\setminus \bar{I}}$ denotes the characteristic function of $\mathbb {T}{\setminus } \bar{I}$, let *v* be a harmonic conjugate of *u*, and set $g \mathrel {\mathop :}=\textrm{e}^{u+\textrm{i}v}$. Then $g :\mathbb {D}\rightarrow \mathbb {D}$ is holomorphic such that |g| extends continuously to ${\bar{\mathbb {D}}} {\setminus } E$ and such that |g|=1 on *I* and |g|<1 on $\mathbb {T}\setminus \bar{I}$. Note that in the special case $I = I_+$ we have the explicit formula

$$\begin{aligned} g(\zeta ) = \left( i\frac{1-\zeta }{1+\zeta }\right) ^{-\frac{i}{\pi }}. \end{aligned}$$

For every $z_0 \in \mathbb {D}$, let

$$\begin{aligned} \varphi _{z_0}(\zeta ) \mathrel {\mathop :}=\frac{z_0 - \zeta }{1-{\bar{z}}_0\zeta }. \end{aligned}$$

### Proposition 5

Let $p = (z_0,0) \in \mathbb {D}\times \{0\}$. Let $g :\mathbb {D}\rightarrow \mathbb {D}$ be a holomorphic function such that |g| extends continuously to ${\bar{\mathbb {D}}} {\setminus } E$ and such that |g|=1 on *I* and |g|<1 on $\mathbb {T}\setminus \bar{I}$. Define $\tilde{f}_\nu (\zeta ) \mathrel {\mathop :}=(\zeta , g^\nu (\zeta ))$. For every sequence $\{r_\nu \}_{\nu =1}^\infty \subset (0,1)$ converging to 1 fast enough, and for $\tau _\nu (\zeta ) \mathrel {\mathop :}=r_\nu \zeta ,$ the holomorphic maps $f_\nu :{\bar{\mathbb {D}}} \rightarrow \mathbb {C}^2$ given by $f_\nu = \tilde{f}_\nu \circ \tau _\nu \circ \varphi _{z_0}$ define a sequence of Poletsky discs for *K* centered at *p*.

### Proof

It suffices to consider the case $z_0 = 0$. Recall first that every bounded pseudoconvex Runge neighborhood of *K* contains $\hat{K}$, see, e.g., Theorem 4.3.3 in [5], and that $\hat{K}$ has a neighborhood basis of bounded pseudoconvex Runge domains, see, e.g., Theorem 1.3.8 in [12]. Let $\varOmega \subset \mathbb {C}^2$ be a bounded pseudoconvex Runge domain containing *K*, let *U* be an open neighborhood of *K*, and let ε>0. From the properties of |g| it follows that $\lim _{\nu \rightarrow \infty } |g^\nu \circ \tau _\nu | = 0$ locally uniformly on $\mathbb {D}\cup (\mathbb {T}{\setminus } \bar{I})$, and that, for $\{r_\nu \}_{\nu =1}^\infty$ converging to 1 fast enough, we have $\lim _{\nu \rightarrow \infty } |g^\nu \circ \tau _\nu | = 1$ locally uniformly on *I*. This shows that, for $\nu \in \mathbb {N}$ large enough, the sets $f_\nu ({\bar{\mathbb {D}}})$ will be contained in any given neighborhood of $({\bar{\mathbb {D}}} \times \{0\}) \cup (\bar{I} \times {\bar{\mathbb {D}}}) = \hat{K}$, and thus in Ω. Moreover, if $B \subset \mathbb {T}$ is an open neighborhood of *E* such that $\sigma (B) < \varepsilon$, then, since $|g^\nu \circ \tau _\nu | \rightarrow \chi _I$ uniformly on $\mathbb {T}{\setminus } B$, we have that for $\nu \in \mathbb {N}$ large enough $f_\nu (\mathbb {T}\setminus B) \subset U$. Lastly, $f_\nu (0) = (0, g^\nu (0)) \rightarrow (0,0)$ for $\nu \rightarrow \infty$. □

For every $p \in \hat{K} \setminus K$, we will also explicitly compute the limit $T = \lim _{\nu \rightarrow \infty }(f_\nu )_*G$ with respect to the Poletsky discs $\{f_\nu \}$ constructed above. We first introduce some notation: For every $z_0 \in \mathbb {D}$, let

$$\begin{aligned} g_\mathbb {D}(z_0,\zeta ) = -\log |\varphi _{z_0}(\zeta )| = -\log \left| \frac{\zeta -z_0}{1-{\bar{z}}_0\zeta }\right| \end{aligned}$$

denote the Green’s function for $\mathbb {D}$, and let

$$\begin{aligned} \omega _\mathbb {D}(z_0,\zeta ) = \frac{1-|z_0|^2}{|\zeta -z_0|^2}\,\textrm{d}\sigma (\zeta ) \end{aligned}$$

be the harmonic measure for $\mathbb {D}$. The Green current with respect to $z_0$ is defined as

$$\begin{aligned} \langle G_{z_0},\alpha \rangle = \int _\mathbb {D}g_\mathbb {D}(z_0, \,\cdot ) \alpha \quad \text {for all } \alpha \in \mathcal {E}^{1,1}({\bar{\mathbb {D}}}). \end{aligned}$$

For $z_0 = 0$, we simply write $G = G_0$. With the normalization $d^\textrm{c} = \frac{i}{2\pi }({\bar{\partial }}-\partial )$, the Green–Riesz representation formula states that

$$\begin{aligned} \langle dd^\textrm{c}G_{z_0},u\rangle = \int _\mathbb {T}u(\zeta ) \,\textrm{d}\omega _\mathbb {D}(z_0,\zeta ) - u(z_0) \quad \text {for all } u \in \mathcal {E}({\bar{\mathbb {D}}}), \end{aligned}$$

i.e., $$dd^\textrm{c}G_{z_0} = \omega _\mathbb {D}(z_0,\,\cdot ) - \delta _{z_0}$$.

Let now $p = (z_0,w_0) \in \hat{K} {\setminus } K$. If $p \in \bar{I} \times {\bar{\mathbb {D}}}$ and $f_\nu (\zeta ) =(z_0,\zeta )$, then we simply have $T=(\jmath _{z_0})_*G$, where $\jmath _{z_0} :\mathbb {D}\hookrightarrow \{z_0\} \times \mathbb {D}$ denotes the natural inclusion. If $p \in \mathbb {D}\times \{0\}$, then we first observe that for $g = \textrm{e}^{u+\textrm{i}v}$, $u = -P[\chi _{\mathbb {T}{\setminus } \bar{I}}]$, as constructed above, not only |g| but also *g* extends continuously to ${\bar{\mathbb {D}}} \setminus E$. This is due to the fact that, while the harmonic conjugate *v* of *P*[*f*] might not extend continuously to points of $\mathbb {T}$ at which *f* is continuous, it does extend continuously to any point of $\mathbb {T}$ at which *f* is continuously differentiable (see, e.g., I.E.2 in [6]). It then suffices to prove the following result.

### Proposition 6

Let $p = (z_0,0) \in \mathbb {D}\times \{0\}$. Let $g :\mathbb {D}\rightarrow \mathbb {D}$ be a holomorphic function such that *g* extends continuously to ${\bar{\mathbb {D}}} {\setminus } E$ and such that |g|=1 on *I* and |g|<1 on $\mathbb {T}\setminus \bar{I}$. Define $\tilde{f}_\nu (\zeta ) \mathrel {\mathop :}=(\zeta , g^\nu (\zeta ))$. For every sequence $\{r_\nu \}_{\nu =1}^\infty \subset (0,1)$ converging to 1 fast enough, and for $\tau _\nu (\zeta ) \mathrel {\mathop :}=r_\nu \zeta ,$ the holomorphic maps $f_\nu :{\bar{\mathbb {D}}} \rightarrow \mathbb {C}^2$ given by $f_\nu = \tilde{f}_\nu \circ \tau _\nu \circ \varphi _{z_0}$ have the property that the sequence $\{(f_\nu )_*G\}$ converges weakly to

$$\begin{aligned} T \mathrel {\mathop :}=(\imath _0)_*G_{z_0} + \int _I (\jmath _\zeta )_*G \,\textrm{d}\omega _\mathbb {D}(z_0,\zeta ), \end{aligned}$$

where $\imath _0 :\mathbb {D}\hookrightarrow \mathbb {D}\times \{0\}$ and $\jmath _\zeta :\mathbb {D}\hookrightarrow \{\zeta \} \times \mathbb {D}$ are the natural inclusions, i.e.,,

$$\begin{aligned} \langle T,\alpha \rangle&= \int _{\mathbb {D}\times \{0\}} g_\mathbb {D}(z_0,\,\cdot ) \alpha \\&+\int _I \left( \int _{\{\zeta \} \times \mathbb {D}} g_\mathbb {D}(0,\,\cdot ) \alpha \right) \textrm{d}\omega _\mathbb {D}(z_0,\zeta ), \quad \alpha \in \mathcal {E}^{1,1}(\mathbb {C}^2). \end{aligned}$$

We first need the following auxiliary statement. The result is certainly well known, but due to the lack of a good reference, we include a proof for the convenience of reading.

### Lemma 7

Let $\mu \ll \sigma$ be a finite Borel measure on $\mathbb {T}$. If $p_\nu (\zeta ) \mathrel {\mathop :}=\zeta ^\nu ,$ $\nu \in \mathbb {N},$ then $\lim _{\nu \rightarrow \infty } (p_\nu )_*\mu = \mu (\mathbb {T})\sigma$ weakly.

### Proof

Since $\mu \ll \sigma$, there exists $a \in L^1(\mathbb {T},\sigma )$ such that $\textrm{d}\mu = a\,\textrm{d}\sigma$, and since trigonometric polynomials are dense in $L^1(\mathbb {T},\sigma )$, there exists $\{a_n\}_{n\in \mathbb {Z}} \subset \mathbb {C}$ such that $a(\zeta ) = \sum _{n=-\infty }^\infty a_n\zeta ^n$ with convergence in $L^1$-norm. Hence, for every $k \in \mathbb {Z},$

$$\begin{aligned}&\int _{\mathbb {T}} \zeta ^k\,\textrm{d}((p_\nu )_*\mu ) = \int _{\mathbb {T}} \zeta ^{k\nu }a(\zeta )\,\textrm{d}\sigma = \sum _{n=-\infty }^\infty \int _{\mathbb {T}}a_n\zeta ^{n+k\nu }\,\textrm{d}\sigma \\&\quad = a_{-k\nu } \rightarrow \left\{ \begin{array}{ccl} a_0 &  , &  k = 0, \\ 0 &  , &  k \ne 0, \end{array} \right. \end{aligned}$$

which shows that $\lim _{\nu \rightarrow \infty } (p_\nu )_*\mu$ and $a_0\sigma$ coincide on the set of all trigonometric polynomials. Appealing again to the density of trigonometric polynomials in $L^1(\mathbb {T},\sigma )$, and observing that $\mu (\mathbb {T}) = \sum _{n=-\infty }^\infty \int _\mathbb {T}a_n\zeta ^n \,\textrm{d}\sigma = a_0$, the statement follows. □

### Proof of Proposition 6

Fix $\{r_\nu \}_{\nu =1}^\infty \subset (0,1)$ converging to 1 so fast that $g^\nu \circ \tau _\nu - g^\nu \rightarrow 0$ for $\nu \rightarrow \infty$ locally uniformly on $\mathbb {T}\setminus E$. Note that this is always possible, since for every ε>0 and every $\nu \in \mathbb {N}$ there exists $\delta _\nu > 0$ such that $|p^\nu -q^\nu | < \varepsilon$ for every $p,q \in {\bar{\mathbb {D}}}^2$ satisfying $|p-q| < \delta _\nu$, and since, by assumption on *g*, for every compact set $L \subset \mathbb {T}{\setminus } E$ and every $r_\nu \in (0,1)$ close enough to 1 we have $|g \circ \tau _\nu - g| < \delta _\nu$ on *L*.

Let $T_\nu \mathrel {\mathop :}=(f_\nu )_*G$, $\nu \in \mathbb {N}$, and observe that it suffices to show that

$$\begin{aligned} \lim _{\nu \rightarrow \infty }\langle T_\nu , dd^\textrm{c} u\rangle = \langle T, dd^\textrm{c} u\rangle \quad \text {for all } u \in \mathcal {E}(\mathbb {C}^2). \end{aligned}$$

Fix $u \in \mathcal {E}(\mathbb {C}^2)$, and without loss of generality assume that $u \not \equiv 0$ on $\bar{\mathbb {D}}^2$. Then

1 $$\begin{aligned} \langle T,dd^\textrm{c}u\rangle&= \langle (\imath _0)_*G_{z_0}, dd^\textrm{c}u\rangle + \int _I \langle (\jmath _\zeta )_*G, dd^\textrm{c} u\rangle \,\textrm{d}\omega _\mathbb {D}(z_0,\zeta ) \nonumber \\&= \int _\mathbb {T}(u \circ \imath _0)(\zeta ) \,\textrm{d}\omega _\mathbb {D}(z_0,\zeta ) - (u \circ \imath _0)(z_0) \nonumber \\&\quad + \int _I \left( \int _\mathbb {T}(u \circ \jmath _\zeta ) \,\textrm{d}\sigma - (u \circ \jmath _\zeta )(0) \right) \textrm{d}\omega _\mathbb {D}(z_0,\zeta ) \nonumber \\&= \int _{\mathbb {T}\setminus I} (u \circ \imath _0)(\zeta ) \,\textrm{d}\omega _\mathbb {D}(z_0,\zeta ) \nonumber \\&\quad + \int _I \left( \int _\mathbb {T}(u \circ \jmath _\zeta ) \,\textrm{d}\sigma \right) \textrm{d}\omega _\mathbb {D}(z_0,\zeta ) - (u \circ \imath _0)(z_0). \end{aligned}$$

On the other hand, we have

2 $$\begin{aligned} \langle T_\nu ,dd^\textrm{c}u\rangle&= \int _\mathbb {T}(u \circ f_\nu )(\zeta )\,\textrm{d}\sigma (\zeta ) - (u \circ f_\nu )(0) \nonumber \\&= \int _\mathbb {T}(u \circ \tilde{f}_\nu \circ \tau _\nu )(\zeta )\,\textrm{d}\omega _\mathbb {D}(z_0,\zeta ) - (u \circ \tilde{f}_\nu \circ \tau _\nu )(z_0), \end{aligned}$$

where in the last equation we have used that $(\varphi _{z_0})_*\sigma = \omega _\mathbb {D}(z_0,\,\cdot )$ (note that $((\varphi _{z_0})_*\sigma )(M) = \omega _\mathbb {D}(0,\varphi _{z_0}^{-1}(M)) = \omega _\mathbb {D}(z_0,M)$ for every Borel set $M \subset \mathbb {T}$, see, e.g., Theorem 4.3.8 in [9]). Let ε>0. Clearly, for $\nu \in \mathbb {N}$ large enough, we have

3 $$\begin{aligned} |(u \circ \tilde{f}_\nu \circ \tau _\nu )(z_0) - (u \circ \imath _0)(z_0)| < \varepsilon . \end{aligned}$$

Since $|g \circ \tau _\nu | \rightarrow |g| < 1$ for $\nu \rightarrow \infty$ locally uniformly on $\mathbb {T}\setminus \bar{I}$, it follows that $\tilde{f}_\nu \circ \tau _\nu \rightarrow \imath _0$ for $\nu \rightarrow \infty$ locally uniformly on $\mathbb {T}\setminus \bar{I}$. Since $f_\nu (\mathbb {D}) \subset \mathbb {D}^2$ for all $\nu \in \mathbb {N}$, it thus follows from uniform continuity of *u* on ${\bar{\mathbb {D}}}^2$ that for $\nu \in \mathbb {N}$ large enough

4 $$\begin{aligned}&\left| \int _{\mathbb {T}\setminus \bar{I}} (u \circ \tilde{f}_\nu \circ \tau _\nu )(\zeta ) \,\textrm{d}\omega _\mathbb {D}(z_0,\zeta )\right. \nonumber \\&\quad \left. -\int _{\mathbb {T}\setminus \bar{I}} (u \circ \imath _0)(\zeta ) \,\textrm{d}\omega _\mathbb {D}(z_0,\zeta ) \right| < \varepsilon . \end{aligned}$$

Fix δ>0 such that |u(p)-u(q)|<ε for all $p,q \in {\bar{\mathbb {D}}}$ with |p-q|<δ. Choose pairwise disjoint, open, connected sets $I_1, \ldots , I_N \subset \subset I$ such that, for every k=1,…,N, one has $|\zeta -\zeta '| < \delta /2$ for $\zeta ,\zeta ' \in I_k$, and such that for $B \mathrel {\mathop :}=I {\setminus } \bigcup _{k=1}^N I_k$ we have $\omega _\mathbb {D}(z_0,B) < (\sup _{{\bar{\mathbb {D}}}^2}|u|)^{-1}\varepsilon$. Note that, in particular, the last condition implies that

5 $$\begin{aligned}&\left| \int _{B} (u \circ \tilde{f}_\nu \circ \tau _\nu )(\zeta ) \,\textrm{d}\omega _\mathbb {D}(z_0,\zeta )\right. \nonumber \\&\quad \left. -\int _{B}\left( \int _\mathbb {T}(u \circ \jmath _{\zeta }) \,\textrm{d}\sigma \right) \,\textrm{d}\omega _\mathbb {D}(z_0,\zeta ) \right| < 2\varepsilon . \end{aligned}$$

Fix points $\zeta _k \in I_k$, k=1,…,N, that will be further specified later on. By the choice of $\{r_\nu \}_{\nu =1}^\infty$, we know that $g^\nu \circ \tau _\nu - g^\nu \rightarrow 0$ for $\nu \rightarrow \infty$ locally uniformly on $\mathbb {T}{\setminus } E$. Thus, for $\nu \in \mathbb {N}$ large enough, we have

$$\begin{aligned}&|(\tilde{f}_\nu \circ \tau _\nu )(\zeta ) - \jmath _{\zeta _k}(g^\nu (\zeta ))| = |(\tau _\nu (\zeta ), (g^\nu \circ \tau _\nu )(\zeta )) \\&\quad - (\zeta _k, g^\nu (\zeta ))| < \delta \quad \text {for all } \zeta \in I_k. \end{aligned}$$

Hence, for every k=1,…,N,

$$\begin{aligned}&\left| \int _{I_k} (u \circ \tilde{f}_\nu \circ \tau _\nu )(\zeta ) \,\textrm{d}\omega _\mathbb {D}(z_0,\zeta )\right. \\&\quad \left. -\int _{I_k} (u \circ \jmath _{\zeta _k})(g^\nu (\zeta )) \,\textrm{d}\omega _\mathbb {D}(z_0,\zeta ) \right| < \omega _\mathbb {D}(z_0,I_k)\varepsilon . \end{aligned}$$

With $p_\nu (\zeta ) \mathrel {\mathop :}=\zeta ^\nu$ and $\mu \mathrel {\mathop :}=g_*(\omega _\mathbb {D}(z_0,\,\cdot )_{|I_k})$, we have $\int _{I_k} (u \circ \jmath _{\zeta _k})(g^\nu (\zeta )) \,\textrm{d}\omega _\mathbb {D}(z_0,\zeta ) = \langle (p_\nu )_*\mu , u \circ \jmath _{\zeta _k}\rangle$, and since we know from Lemma 7 that $\lim _{\nu \rightarrow \infty } (p_\nu )_*\mu = \mu (\mathbb {T}) \sigma = \omega _\mathbb {D}(z_0,I_k)\sigma$ weakly, it follows that, for $\nu \in \mathbb {N}$ large enough,

$$\begin{aligned} \left| \int _{I_k} (u \circ \jmath _{\zeta _k})(g^\nu (\zeta )) \,\textrm{d}\omega _\mathbb {D}(z_0,\zeta ) -\omega _\mathbb {D}(z_0,I_k)\int _\mathbb {T}(u \circ \jmath _{\zeta _k}) \,\textrm{d}\sigma \right| < \textstyle \frac{1}{N}\varepsilon . \end{aligned}$$

Lastly, since $\omega _\mathbb {D}(z_0,\,\cdot )$ is absolutely continuous with respect to σ, by the mean value theorem we can choose the points $\zeta _k \in I_k$ in such a way that, for every k=1,…,N,

$$\begin{aligned} \omega _\mathbb {D}(z_0,I_k)\int _\mathbb {T}(u \circ \jmath _{\zeta _k})\,\textrm{d}\sigma = \int _{I_k}\left( \int _\mathbb {T}(u \circ \jmath _{\zeta }) \,\textrm{d}\sigma \right) \textrm{d}\omega _\mathbb {D}(z_0,\zeta ). \end{aligned}$$

Combining the last three displayed formulas, and summing up over *k*, we obtain, with $I' \mathrel {\mathop :}=\bigcup _{k=1}^N I_k$,

6 $$\begin{aligned}&\left| \int _{I'} (u \circ \tilde{f}_\nu \circ \tau _\nu )(\zeta ) \,\textrm{d}\omega _\mathbb {D}(z_0,\zeta )\right. \nonumber \\&\quad \left. -\int _{I'}\left( \int _\mathbb {T}(u \circ \jmath _{\zeta }) \,\textrm{d}\sigma \right) \textrm{d}\omega _\mathbb {D}(z_0,\zeta ) \right| < 2\varepsilon . \end{aligned}$$

Recalling the Formulas (1) and (2) for $\langle T, dd^\textrm{c}u\rangle$ and $\langle T_\nu , dd^\textrm{c}u\rangle$, we see that the estimates (3)–(6) show that

$$\begin{aligned} |\langle T_\nu ,dd^\textrm{c}u\rangle - \langle T,dd^\textrm{c}u\rangle | < 6\varepsilon \end{aligned}$$

for $\nu \in \mathbb {N}$ large enough, which proves the claim. □ □

### Remark

Note that (1) shows that $dd^\textrm{c}T = {\tilde{\sigma }} - \delta _p$ with

$$\begin{aligned} {\tilde{\sigma }} = \left( \omega _\mathbb {D}(z_0,\,\cdot )_{|\mathbb {T}\setminus I} \times \delta _0\right) + \left( \omega _\mathbb {D}(z_0,\,\cdot )_{|I} \times \sigma \right) , \end{aligned}$$

and clearly ${\tilde{\sigma }}$ is a representative Jesen measure for *p*.

## Data Availability

No datasets were generated or analyzed during the current study.
